# Development of an ERA-CRISPR/Cas12a-based visual diagnostic assay for bovine babesiosis

**DOI:** 10.3389/fvets.2026.1917943

**Published:** 2026-08-21

**Authors:** Yongchang Li, Xiujuan Feng, Xianyue Fu, Fakiha Kalim, Shiyun Li, Iqra Zafar, Mohamed Abdo Rizk, Zeyun Cui, Bayin Chahan, Yinke Li, Dongpo Ning, Qingyong Guo

**Affiliations:** 1 Parasitology Laboratory, Veterinary College, Xinjiang Agricultural University, Urumqi, Xinjiang, China; 2 Laboratory of Sustainable Animal Environment, Graduate School of Agriculture Science, Tohoku University, Sendai, Japan; 3 Department of Internal Medicine and Infectious Diseases, Faculty of Veterinary Medicine, Mansoura University, Mansoura, Egypt; 4 Department of Clinical Science, College of Veterinary Medicine, King Faisal University, Al-Hofuf, Al-Ahsa, Saudi Arabia

**Keywords:** Babesia bovis, CRISPR Cas12a, enzymatic recombinase amplification, lateral flow strips, molecular detection, spherical body proteins

## Abstract

Bovine babesiosis, caused by *Babesia bovis*, is a tick-borne hemoparasitic disease causing substantial economic losses to cattle industries worldwide. Current diagnostic methods, particularly microscopy, lack sensitivity for subclinical infections, while molecular assays require sophisticated equipment unsuitable for field use. To address this gap, we developed a novel diagnostic assay integrating enzymatic recombinase amplification (ERA) with CRISPR-Cas12a-mediated trans-cleavage, targeting the *B. bovis* spherical body protein 2 (SBP2) gene. The assay enables visual readout via UV/blue light fluorescence and lateral flow strips. Following systematic optimization of primers, crRNA, reaction temperature, and reporter concentration, the ERA-CRISPR/Cas12a system achieved complete amplification within 20 min at 37 °C and demonstrated a detection limit of 10 copies/μL with high specificity for *B. bovis*. comparable diagnostic performance with conventional PCR within the tested sample panel. The fluorescence readout achieved a detection limit of 10^1^ copies/μL, while the lateral-flow strip format exhibited a detection limit of 104 copies/μL. The entire procedure, from DNA extraction (approximately 30 min) to final result readout (ERA: 20 min; CRISPR-Cas12a: 20 min), can be completed within approximately 70 min. This detection method supports two types of visual detection: portable blue light and test strips, and is suitable for on-site environments with limited resources. This work provides a practical diagnostic tool for rapid, accurate detection of bovine babesiosis, with potential applicability to other veterinary pathogens.

## 1 Introduction

Bovine babesiosis, caused by apicomplexan parasites of the genus *Babesia*, including *Babesia bigemina, Babesia ovata, Babesia bovis*, is a tick-transmitted hemoprotozoan disease that imposes a heavy burden on cattle production across tropical and subtropical regions worldwide (1). Following erythrocyte invasion, the parasite triggers fever, anemia, and hemoglobinuria; in its acute form, the disease frequently proves fatal, inflicting severe economic damage on the livestock sector (2). The infection is highly prevalent throughout Africa, Australia, and large parts of Central and South America. In China, it has been reported in 21 provinces and municipalities- including Gansu, Qinghai, and Xinjiang, hindering the development of the cattle industry. Climate change has further facilitated the northward expansion of tick habitats and amplified the force of infection of tick-borne pathogens (3). Xinjiang, situated at the heart of the Silk Road Economic Belt, serves as a critical gateway for the movement of livestock and personnel between China, Central Asia, and Europe (4). The region has been recognized as a high-risk focus for vector-borne diseases, with indigenous transmission of Crimean-Congo hemorrhagic fever, Q fever, babesiosis, and Lyme disease (5). Notably, in 2020, Kazakhstan—one of the eight countries bordering China's “Belt and Road” initiative—reported an outbreak of a tick-borne disease (6). The diverse ecosystems of Xinjiang, which include forests, shrublands, grasslands, and semi-desert areas, provide favorable conditions for tick proliferation (34).

Current diagnostic methods for babesiosis include microscopic examination, serological assays such as ELISA and indirect fluorescent antibody tests, and molecular techniques like conventional PCR, nested PCR, and qPCR [(7, 37)]. Isothermal platforms such as LAMP (Loop-mediated isothermal amplification) have also been explored (8). However, microscopy and serological tests suffer from low sensitivity, high labor requirements, and a tendency for missed diagnoses, rendering them unsuitable for early detection. PCR-based approaches, while markedly more sensitive, depend on expensive thermal cyclers, require lengthy processing times, and necessitate post-amplification electrophoresis, rendering them impractical for on-site application in resource-limited settings (3). While LAMP is isothermal, it involves extended reaction times (60–70 min), strict temperature control (~65 °C), complex primer design, and a high false-positive rate, making it less practical than alternative isothermal techniques such as RPA and ERA. Diagnostic delays not only accelerate disease dissemination but also increase the likelihood of species misidentification, which may prompt inappropriate chemotherapy and, consequently, fuel the emergence of drug resistance (9). To circumvent these obstacles, the present study endeavors to engineer a rapid, highly sensitive, and field-compatible point-of-care testing (POCT) platform to strengthen early warning systems and epidemiological surveillance for bovine babesiosis.

ERA (Enzymatic Recombinase Amplification) is a streamlined, innovative method for isothermal amplification of nucleic acids that functions optimally at a constant temperature (37–42 °C), without requiring denaturation, annealing, or thermal cycling steps—thereby completely obviating the need for a thermocycler (10). Simultaneously, the CRISPR-Cas system, particularly the Cas12a protein, provides exceptional specificity and powerful signal amplification. Upon sequence-specific recognition of its DNA target, Cas12a unleashes non-specific trans-cleavage of single-stranded DNA reporters, enabling simple visual readout via fluorescence emission or lateral-flow dipsticks (11). ERA-based assays have already been developed for several important infectious agents, including infectious hypodermal and hematopoietic necrosis virus (IHHNV), hepatitis B virus, and Plasmodium spp (12–14). In recent years, CRISPR-Cas-based diagnostic platforms have emerged as powerful tools for detecting various veterinary pathogens. For example, RPA-CRISPR/Cas12a assays have been developed for the detection of *Hepatozoon canis* in dogs, and *B. bigemina* (15, 16). Additionally, CRISPR-Cas12a-based pen-side tests have been successfully established for *Theileria parva* infections in cattle, demonstrating the versatility of this platform for diagnosing tick-borne diseases (17).

This study integrates the high efficiency of ERA amplification with the CRISPR-Cas12a system to establish a detection method based on the coupling of ERA and CRISPR-Cas12a technology, which exhibits high sensitivity and specificity. This approach enables, for the first time, visual detection of *B. bovis*. The developed method provides a practical technical approach for the rapid on-site detection, epidemic monitoring, and control of *B. bovis*. Although CRISPR-based diagnostics have been established for several tick-borne pathogens, no ERA-CRISPR/Cas12a assay specifically targeting *the B. bovis SBP2* gene has been reported to date. The present study therefore introduces a novel application of this technology by combining the high amplification efficiency of ERA with the trans-cleavage activity of Cas12a for *B. bovis* detection. Furthermore, we provide a comparative evaluation of fluorescence and lateral-flow readouts to accommodate different field scenarios, which represents a practical advancement over single-format assays.

## 2 Materials and methods

Positive genomic DNA samples for the listed pathogens, *B. bovis* (*Bbov*), *Babesia microti* (*Bm*), *Theileria annulata* (*Tann*), *Anaplasma marginale* (*Anap*), *Rickettsia spp*. (*Rick*), and *Trypanosoma evansi* (*Teva*), were supplied by the Parasitology Laboratory of the College of Veterinary Medicine, Xinjiang Agricultural University. All primers, ssDNA and crRNA used in this study were synthesized by Sangon Biotech (Shanghai, China) and the sequences were shown in Supplementary Table 1. In brief, the Basic Enzymatic Recombinase Amplification, PMD 19-T vector, DNA extraction reagents, 2× Taq Master Mix, agarose gel recovery kit, DNA purification kit were the same as those used in our previous study (18). Cas12a and its corresponding NEBuffer r2.1 were purchased from New England Biolabs (MA, USA). CRISPR-based lateral flow strips were purchased from Warbio Biological Co., Ltd. (cat. no. JY0301). Plasmid copy number was calculated according to the following formula: Copy number (copies/μL) = [DNA concentration (g/μL)/(plasmid length in base pairs × 660)] × 6.02 × 10^23^. The purified plasmid was serially diluted with 1× TE buffer to final concentrations spanning 10^6^ to 10^0^ copies/μL, aliquoted as standard solutions, and stored at −20 °C until use. Three primer pairs targeting conserved regions of the *B. bovis*-*SBP2* were designed using Primer Premier 5.0. These were screened to identify the pair with optimal amplification efficiency and specificity. Two crRNAs were subsequently designed using the CRISPR online design tool (http://crispor.org/), targeting the amplicon internal region flanked by the selected primer pair. For fluorescence-based detection, the ssDNA of the fluorescence detection system is modified by FAM and BHQ, while for the lateral flow band (LFS) detection system, the modification of ssDNA is replaced by FAM and Biotin. All oligonucleotide sequences are listed in Supplementary Table 1.

The assay employed a two-stage workflow: (1) amplification of the *SBP2* gene fragment using the Basic ERA kit, followed by (2) combining the ERA product with the CRISPR system. Detailed procedural steps are described in our previous work (18). To fulfill the requirement for detailed optimization, we systematically evaluated key parameters. For ERA, temperatures (35, 37, 39, 41 °C) and durations (10, 15, 20, 30 min) were tested, with 37 °C for 20 min yielding the highest amplification efficiency. Primer concentrations ranging from 0.2 to 1.0 μM were assessed, and 0.5 μM (2.5 μL of 10 μM stock) was selected to minimize primer-dimer formation. For the CRISPR-Cas12a cleavage, the Cas12a:crRNA molar ratio was optimized at 1:1, 1:1.5, and 1:2; the ratio 1:1.5 and incubation at 37 °C for 25 min produced the maximal fluorescent signal., The template volume added to ERA was varied from 0.5 to 8.0 μL; 2.0 μL (approximately 10^4^ copies based on standard curve) was chosen as optimal for routine detection, providing clear positive-negative discrimination. For assay evaluation, a panel of 16 well-characterized bovine blood samples (4 positive and 12 negative for *B. bovis*, confirmed by PCR) and 7 spiked samples with known template concentrations (10^0^-10^6^ copies per reaction) were used to determine the limit of detection and specificity. For LFS-based visualization, the FAM-Biotin dual-labeled reporter was substituted for the FAM-BHQ in the CRISPR reaction. Following completion of the CRISPR/Cas12a cleavage step, 5 μL of the reaction mixture was mixed with 45 μL of nuclease-free water. The test strip was then inserted and the result was observed following a 2-min incubation at room temperature. A series of parameters were systematically optimized to maximize assay performance, including: (a) ERA primer pair selection; (b) reaction time and temperature; (c) crRNA sequence; (d) Cas12a:crRNA molar ratio; and (e) ssDNA reporter concentration. All optimization experiments were conducted using the 10^6^ copies/μL plasmid standard as the template. For gel electrophoresis validation, ERA amplicons were purified by adding an equal volume of phenol-chloroform, followed by centrifugation at 12,000 rpm for 5 min; the supernatant was then used for gel electrophoresis. The FAM-Biotin ssDNA concentration was evaluated across a 10–80 nM range to identify the optimal working concentration. During this optimization, varying concentrations of the ssDNA were added to nuclease-free water, and test strips were inserted to monitor C-line development intensity. To assess specificity, genomic DNA from phylogenetically related or clinically confusable hemoparasites—namely *B. microti* (*Bm*), *Theileria annulata* (*Tann*), *Anaplasma marginale* (*Anap*), *Rickettsia spp*. (*Rick*), and *Trypanosoma evansi* (*Teva*)—was tested in parallel using both the fluorescence and LFS detection formats. For sensitivity determination, the serially diluted *SBP2* plasmid standards (10^6^ to 10^0^ copies/μL) were subjected to the optimized ERA-CRISPR/Cas12a assay to establish the limit of detection (LOD). Genomic DNA extracted from bovine whole-blood specimens was analyzed in parallel by conventional PCR (with agarose gel electrophoresis) and the established ERA-CRISPR/Cas12a method. The optimized reaction system consisted of: 2 μL NEBuffer r2.1, 200 nM Cas12a (1 μM), 250 nM crRNA (1 μM), 2 μL ERA product, 1,000 nM ssDNA (F–Q), 20 nM ssDNA (F–B), and nuclease-free water was added to a final volume of 20 μL. Experimental results were analyzed as follows, fluorescence values were quantified using ImageJ. Statistical analysis was performed with IBM SPSS Statistics 27. Each experiment was performed in at least three independent replicates, with data expressed as mean ± standard error (SEM).

Statistical analyses were performed using IBM SPSS Statistics 26.0. All fluorescence intensity data were expressed as mean ± standard deviation (SD) from three independent experimental replicates. Given the extremely small sample size per group (*n* = 3), formal Shapiro–Wilk normality test and Levene's homogeneity-of-variance test lack sufficient statistical power and reliable inference, so these tests were not conducted in the present study. Unpaired two-tailed Student's *t*-test and one-way ANOVA followed by Tukey's post-hoc test were adopted for intergroup comparisons based on common practice in similar molecular diagnostic assays with limited replicates. Statistical significance was defined as *P* < 0.05, ^**^P < 0.01, ^***^P < 0.001, ^****^P < 0.0001.

## 3 Results

**Figure 1:**
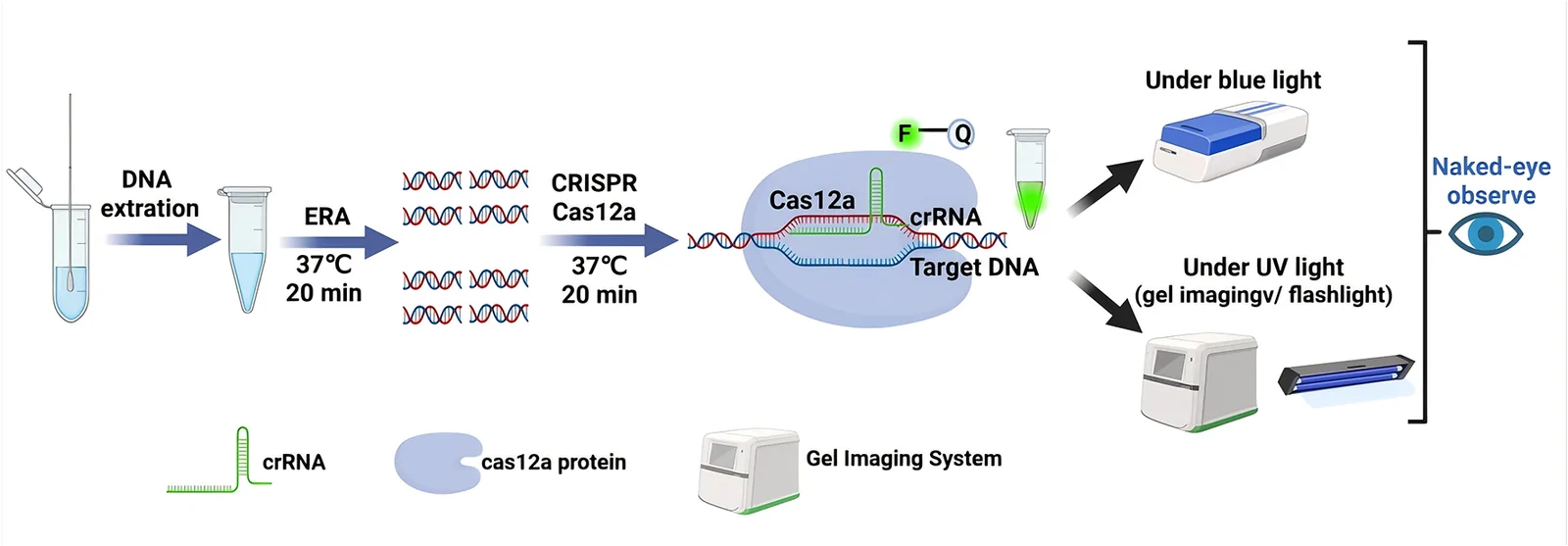
Schematic workflow of the ERA-CRISPR/Cas12a assay for *Babesia bovis* detection. The procedure comprises two main steps. First, genomic DNA extracted from bovine blood samples is subjected to isothermal amplification using the Enzymatic Recombinase Amplification (ERA) system at 37 °C for 20 min, targeting the *SBP2* gene of *B. bovis*. Second, the ERA amplicon is mixed with the CRISPR-Cas12a detection mixture containing crRNA, Cas12a protein, and a fluorescent ssDNA reporter, followed by incubation at 37 °C for 20 min. Upon specific recognition of the target amplicon by the crRNA-Cas12a complex, the trans-cleavage activity of Cas12a is activated, resulting in cleavage of the ssDNA reporter. The results can be interpreted via three independent readout modalities: fluorescence signal detection under UV light using a gel imaging system or a handheld flashlight, fluorescence visualization under blue light, and naked-eye observation of lateral flow strip (LFS) results. The entire process, from DNA extraction to result readout, can be completed within approximately 70 min.

**Figure 2:**
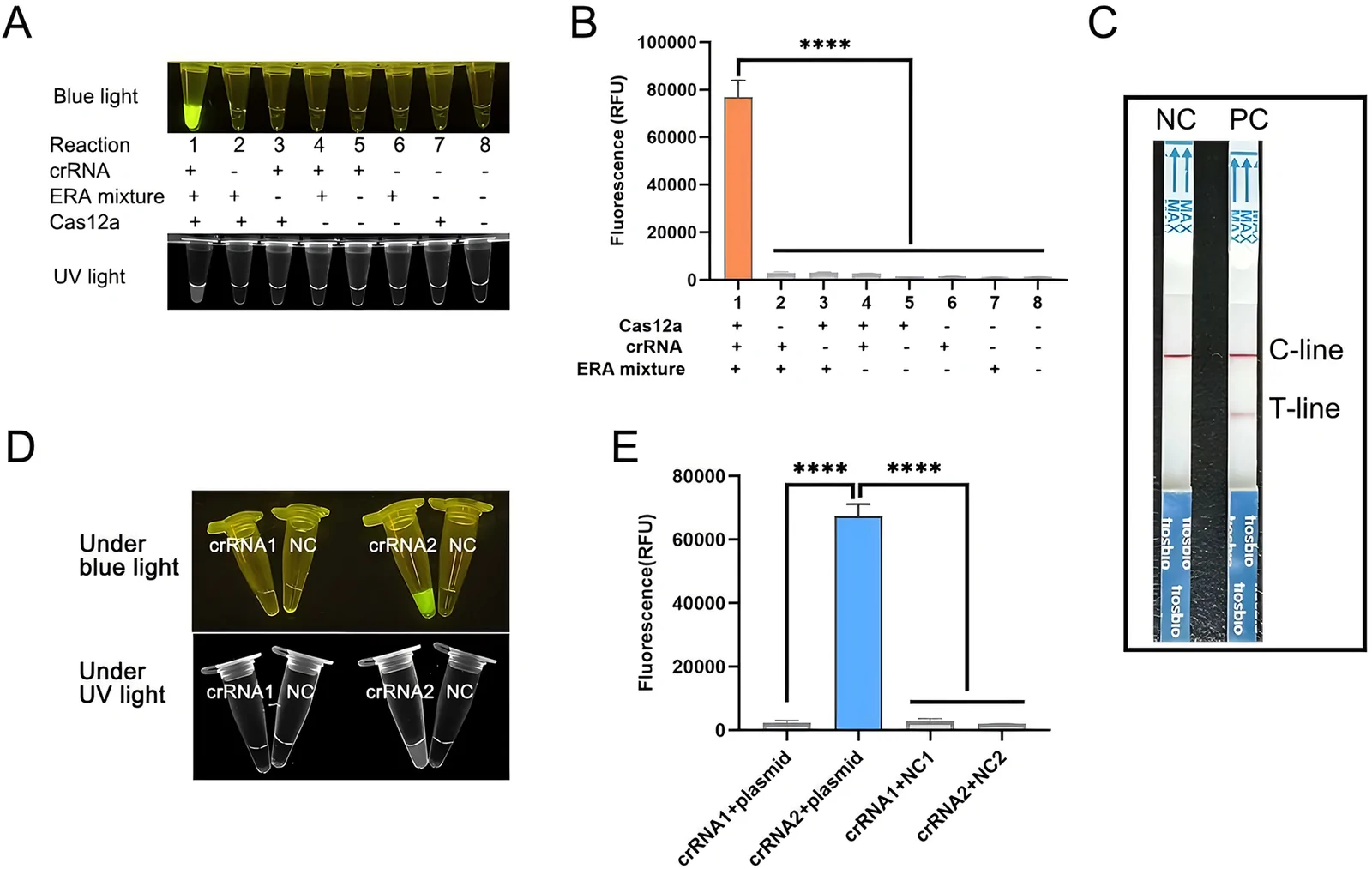
Establishment and validation of the ERA-CRISPR/Cas12a detection system. **(A)** Photographs of reaction tubes under blue light and UV light, showing visible fluorescence signals in the positive control (PC) whereas the negative control (NC) remains dark. **(B)** Quantitative fluorescence intensity (RFU) measured for PC and NC groups, demonstrating a statistically significant difference (****P < 0.0001; unpaired t-test). Data are presented as mean ± SD (*n* = 3). **(C)** Lateral flow strip (LFS) results for PC and NC samples. The presence of both a test line (T-line) and control line (C-line) indicates a positive result, whereas appearance of the C-line only denotes a negative result. **(D, E)** Comparison of two crRNA candidates (crRNA1 and crRNA2) targeting different regions of the *SBP2* gene. Photographs under blue light **(D)** and UV light **(E)** reveal that crRNA2 produces significantly stronger fluorescence signals than crRNA1 when combined with the target plasmid, indicating that crRNA2 possesses superior cleavage efficiency and specificity. NC1 and NC2 represent the corresponding negative controls for each crRNA.

To expedite detection of *B. bovis*, an ERA-CRISPR/Cas12a-based platform was constructed integrating isothermal nucleic acid amplification with CRISPR-mediated signal readout. The experimental workflow is schematically depicted in Figure 1, and proof-of-concept validation is presented in Figures 2A, B. Fluorescence generation required the concurrent presence of all four components: ERA amplification mixture, crRNA, Cas12a protein, and ssDNA reporter. As shown, the absence of any single component resulted in complete loss of signal., The entire assay can be completed within 40 minutes at 37 °C (Setting aside the DNA extraction steps), and results can be visually interpreted using either a portable blue-light transilluminator, UV light and lateral flow strips (Figures 2A, C), making it highly suitable for field deployment.

Three primer sets—F1R1 (180 bp), F2R2 (261 bp), and F3R3 (253 bp)—were screened using ERA-PAGE (Supplementary Figure 1), with amplification compositions detailed in Supplementary Table 2. Electrophoretic banding patterns indicated that F1R1, which generated the shortest amplicon, yielded the brightest product band and was free of non-specific amplification. In contrast, the longer amplicons (F2R2 and F3R3) exhibited either poor amplification efficiency or spurious bands. Therefore, F1R1 was designated as the optimal primer pair for subsequent experiments. Temperature optimization experiments (Supplementary Figure 3) showed that maximal amplification was achieved at 37 °C. To minimize the detection time, 20 min was determined to be the optimal amplification duration (Supplementary Figure 2). Collectively, the optimized ERA conditions were established as 37 °C for 20 min. The entire procedure, from DNA extraction (approximately 30 min) to final result readout (ERA: 20 min; CRISPR-Cas12a: 20 min), can be completed within approximately 70 min.

**Figure 3:**
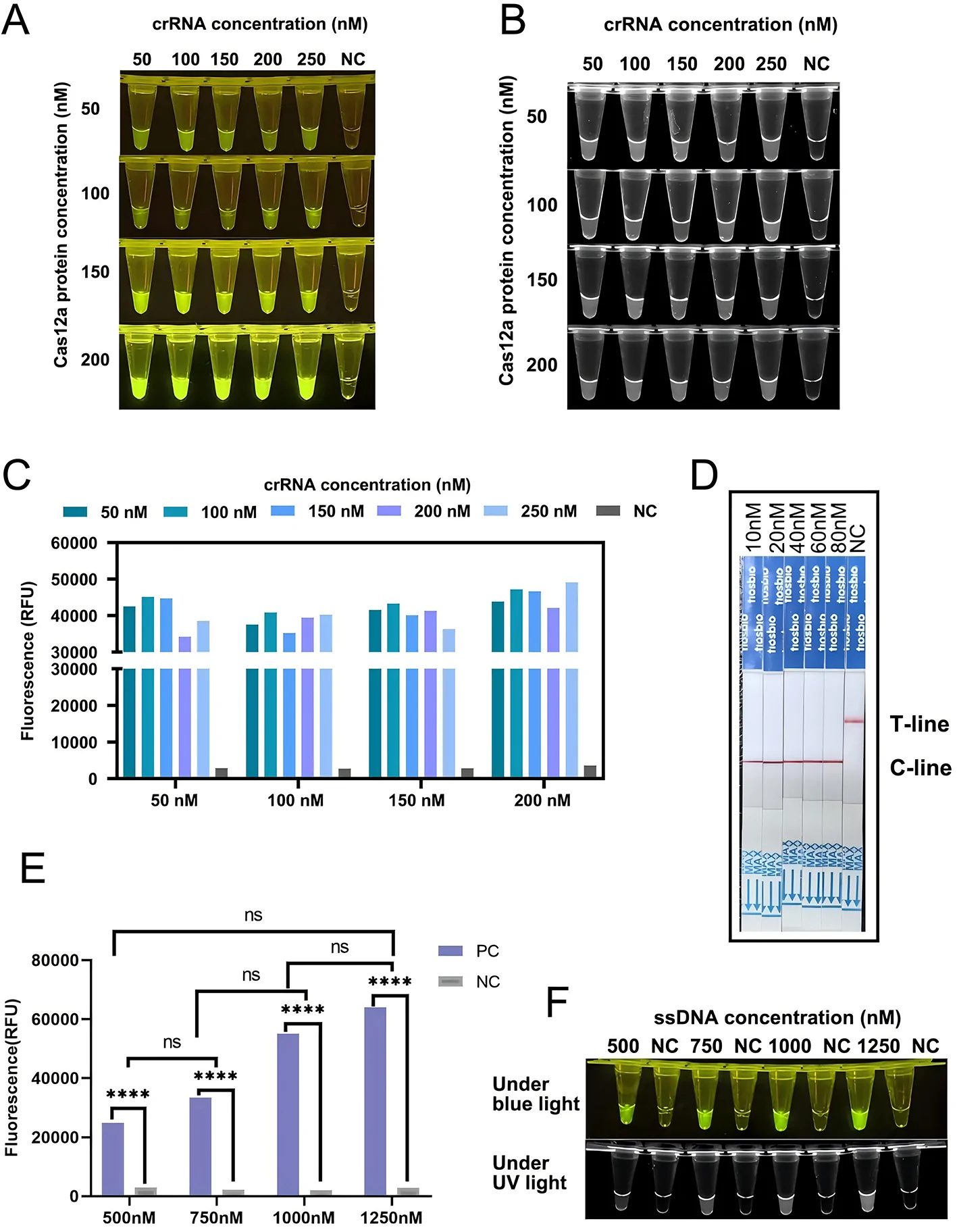
Optimization of key reaction components for the CRISPR-Cas12a detection system. **(A–C)** Optimization of crRNA concentration (50–250 nM) and Cas12a protein concentration (50–200 nM) using fluorescence readout. The combination of 250 nM crRNA and 200 nM Cas12a protein yielded the highest fluorescence signal (RFU) with minimal background in the NC group, and was therefore selected as the optimal working concentration. **(D)** Using the LFS system to optimize the concentration of FAM Biotin single stranded DNA reporter gene, the optimal concentration was screened as 20 nM. **(E)** Optimization of ssDNA reporter concentration (500–1,250 nM) for fluorescence detection. Results demonstrate that increasing reporter concentration significantly enhances fluorescence intensity, with 1,000 nM selected as the optimal concentration (****P < 0.0001; ns, not significant; one-way ANOVA followed by Tukey's multiple comparisons test). **(F)** Photographs under blue light and UV light in the inset further confirm the concentration-dependent fluorescence enhancement. Data are presented as mean ± SD (*n* = 3).

Two candidate crRNAs were screened for their ability to guide the CRISPR-Cas12a system. crRNA2 yielded a robust fluorescence signal, whereas crRNA1 failed to induce detectable activity (Figures 2D, E). Accordingly, crRNA2 was adopted for all subsequent assays. To further refine the system, Cas12a concentrations spanning 50-200 nM and crRNA concentrations spanning 50–250 nM were systematically tested (Figures 3A, B). The strongest signal intensity and fastest reaction kinetics were observed with 200 nM Cas12a and 250 nM crRNA (Figure 3C) and this combination was selected for all further experiments.

The FAM-BHQ ssDNA reporter concentration was titrated across 500–1,250 nM (Figures 3E, F). Although signal intensity rose with increasing reporter concentration, the responses at 1,000 nM and 1,250 nM were nearly equivalent; thus, 1,000 nM was chosen to balance high sensitivity with reagent economy. For LFS-based detection, reporter concentrations were evaluated between 10 and 80 nM. Lower concentrations produced a more intense control (C) line, but a visible test (T) line appeared even in the no-template control (ddH_2_O only) (Figure 3D). likely due to non-specific background binding or competitive displacement effects. Therefore, this study chose a concentration of 20 nM for all subsequent LFS based experiments to ensure clear negative control results.

**Figure 4:**
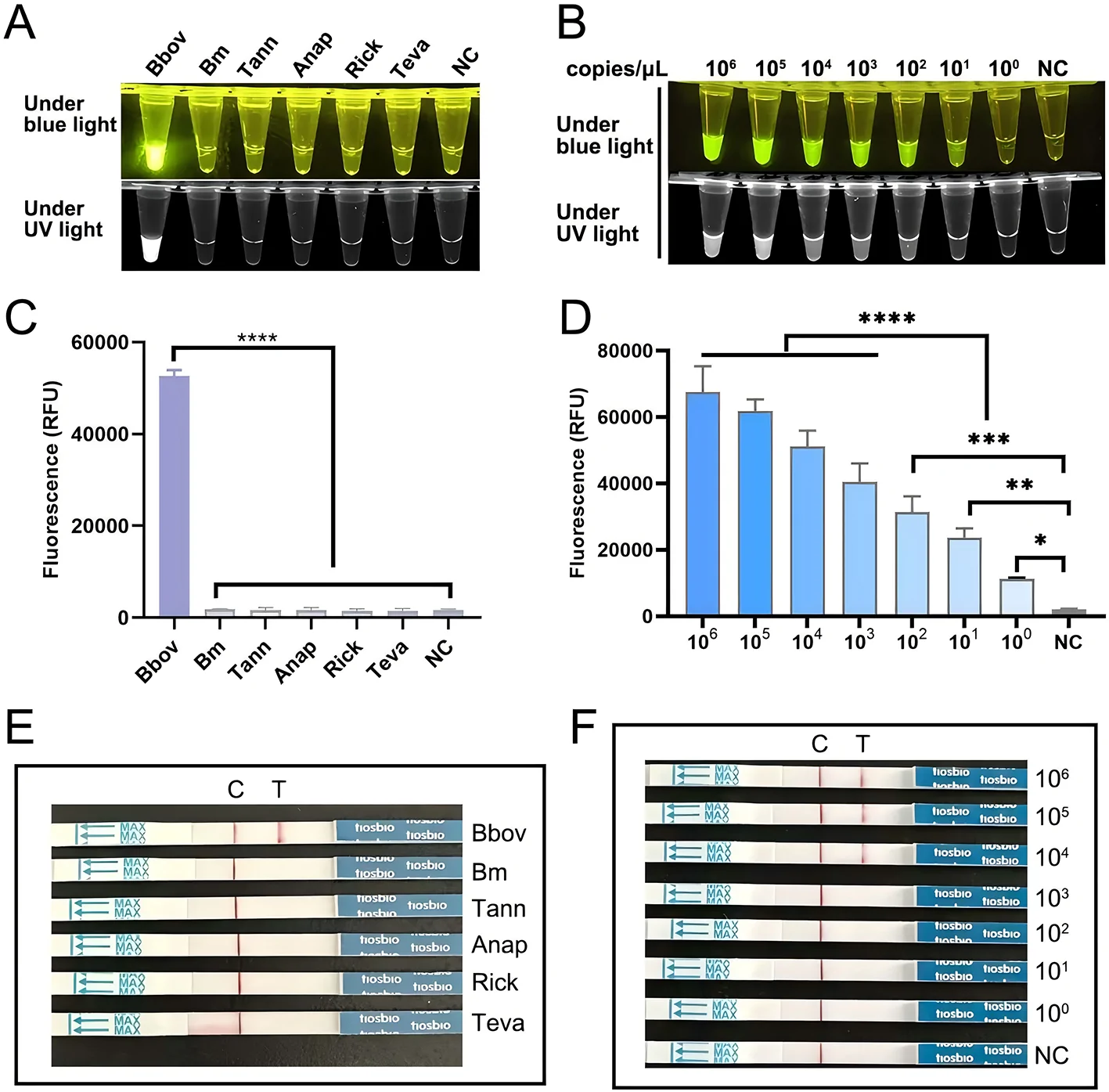
Specificity, sensitivity, and clinical performance of the ERA-CRISPR/Cas12a assay. **(A)** Specificity evaluation using LFS format. The assay specifically detected *B. bovis* (*Bbov*) with clear test lines, whereas no cross-reactivity was observed with other common bovine pathogens, including *B. microti* (*Bm*), *T. annulata* (*Tann*), *A. marginale* (*Anap*), *Rickettsia spp. spp*. (*Rick*), and *T. evansi* (*Teva*). NC, negative control. **(B)** Specificity evaluation using fluorescence readout under blue light, showing positive signals exclusively for *Bbov* samples. **(C, D)** Quantitative fluorescence intensity (RFU) corresponding to the specificity results in **(A)** and **(B)**, confirming the high specificity of the assay (****P < 0.0001; one-way ANOVA). **(E, F)** LFS results for sensitivity testing using 10-fold serial dilutions of the target plasmid (10^6^ to 10° copies/μL). Clear test lines are detectable down to 10^4^ copies/μL, establishing the LFS detection limit. Fluorescence sensitivity testing using the same dilution series, demonstrating that the fluorescence readout can detect as low as 10^1^ copies/μL, indicating a 1,000-fold higher sensitivity compared to the LFS format. NC, no template control. All experiments were performed in triplicate with consistent results.

For the fluorescence format, clear positive signals were detectable down to 10^1^ copies/μL (Figure 4G), establishing the analytical sensitivity of the fluorescence readout. In contrast, the LFS format produced visible test lines only at concentrations of 10^4^ copies/μL and above (Figures 4E, F), indicating a 1,000-fold lower sensitivity compared to the fluorescence readout. Specificity testing using both the fluorescence and LFS methods demonstrated that the assay could accurately distinguish *B. bovis* (*Bbov*) from five other common pathogens: *Babesia microti* (*Bm*), *Theileria annulata* (*Tann*), *Anaplasma marginale* (*Anap*), *Rickettsia spp*. (*Rick*), and *Trypanosoma evansi* (*Teva*), with no observed cross-reactivity (Figures 4A, C, E). To further evaluate the diagnostic performance, a panel of 16 bovine blood DNA samples, collected from clinically suspected cattle in endemic areas, was tested in parallel using our ERA-CRISPR/Cas12a assay (both fluorescence and LFS readouts) and conventional PCR. Among these, 4 samples were confirmed as true-positive for *B. bovis* by conventional PCR (reference method), while the remaining 12 were determined as true-negative, showing no detectable *B. bovis* DNA. The established method correctly identified all 4 truepositive and 12 truenegative samples (Supplementary Figures 5A–D), yielding 100% concordance with conventional PCR (Supplementary Figure 4). This demonstrates that our assay not only possesses high sensitivity and specificity but also exhibits excellent accuracy when applied to clinically relevant specimens.

Based on the 16 clinical samples tested, with conventional PCR as the reference method, the ERA-CRISPR/Cas12a assay demonstrated 100% sensitivity (4/4 true positives) and 100% specificity (12/12 true negatives), yielding a positive predictive value of 100% and a negative predictive value of 100% within this sample set. Cohen's kappa coefficient was 1.00, indicating perfect agreement with conventional PCR. Diagnostic sensitivity and specificity and their corresponding 95% CIs were calculated via the Clopper–Pearson exact binomial method, which is the standard gold-standard approach for small sample datasets and proportions equal to 100%. The core formula of Clopper–Pearson exact CI relies on F-distribution quantiles to compute the lower and upper bounds of binomial proportions. Our clinical 4-fold table data: 4 true positives among 4 pathogen-positive samples, 12 true negatives among 12 pathogen-negative samples. Sensitivity = 4/4 = 100%, 95% CI: 39.76%−100%; Specificity = 12/12 = 100%, 95% CI: 73.54%−100%. The wide confidence intervals clearly reflect the uncertainty caused by limited positive samples. Therefore, given the limited sample size, these values should be interpreted with caution, and larger-scale validation is required to establish robust diagnostic performance metrics.

## 4 Discussion

In the present study, we developed a rapid and sensitive ERA-CRISPR/Cas12a-based diagnostic platform *for B. bovis* detection. The assay is of particular relevance to Xinjiang and similar pastoral regions where livestock production is intensive and tick-borne disease pressures are escalating. Spherical body proteins (SBPs) constitute a protein family that plays an essential role in Babesia invasion of host erythrocytes. Located primarily in the spherical body—a specialized organelle of the parasite—SBPs are involved in host cell invasion and pathogenesis (19). Among the four SBP members (SBP1–SBP4), SBP1, SBP3, and SBP4 are encoded by single-copy genes (20–22). Despite this complexity, *SBP2* remains highly conserved across geographically distinct isolates (23). Previous research has demonstrated that a nested PCR (nPCR) assay targeting the spherical body protein 2 (SBP2) exhibits superior sensitivity for detecting *B. bovis*, achieving a detection rate of 87.6% (127/145). In contrast, an nPCR assay based on the rhoptry-associated protein-1 gene (RAP-1) yielded a significantly lower detection rate of 37.2% (51/145) (20). These observations underscore the advantage of *SBP2* as a diagnostic target, which informed our choice of this gene for assay development.

In recent years, babesiosis prevalence across cattle, equids, and canines in Xinjiang has exhibited a concerning upward trajectory, posing ongoing threats to the livestock economy and public health. Molecular epidemiological investigations over the past three years have revealed a trend toward diversification and complexity of Babesia pathogens in Xinjiang. In cattle, *B. bovis* and *B. bigemina* are relatively common, and seropositivity rates among yaks in certain areas have reached notably high levels (24). In equids, *B. caballi* has also been identified, with evidence suggesting that locally circulating strains may possess unique genotypes that complicate diagnosis (25). As the primary vector, the carrier rate of ticks is crucial for risk assessment. Surveillance data from 2023–2024 indicate a high positive detection rate of Babesia in tick species such as *Rhipicephalus sanguineus* collected from border areas of Xinjiang, highlighting their key role in disease transmission (26). In summary, the current situation in Xinjiang—characterized by multiple hosts, diverse pathogens, and highly active vectors—urgently requires an on-site, rapid, and accurate detection tool to facilitate timely epidemic monitoring and control. The ERA-CRISPR/Cas12a method established in this study is designed to meet this pressing need.

Compared to conventional molecular detection techniques, the core advantage of this method lies in its minimal reliance on complex laboratory conditions and its “user-friendly” operation. Although PCR and quantitative real-time PCR (qPCR) are widely regarded as the gold standard for babesiosis diagnosis, they require precise thermal cyclers, uninterrupted power supply, and trained personnel—conditions often unmet in resource-constrained settings such as local veterinary stations, pastures, or border checkpoints (27, 28). In contrast, the add-incubate-read workflow of our assay substantially reduces the technical expertise required for operation. This aligns well with the need for “simple, rapid, and simple to operate” procedures among frontline animal health workers, providing a powerful tool for field screening and early diagnosis of babesiosis. The ERA/CRISPR-Cas12a platform bypasses thermal cycling altogether, requiring only 20 min of isothermal amplification, and enables highly specific detection via fluorescence or lateral flow strips without dependence on costly instrumentation (29).

Notably, among the three primer sets evaluated (Amplicon sizes: 180 bp, 261 bp, and 253 bp), marked differences in amplification efficiency were observed. The F2R2 (261 bp), and F3R3 (253 bp) primers exhibited non-specific amplification or weak target band signals, likely due to reduced amplification efficiency associated with longer fragments under isothermal conditions. In contrast, the F1R1 primer set, which yields the shortest amplicon (180 bp), demonstrated the highest amplification efficiency, specificity, and reproducibility in the Basic-ERA system and was therefore selected as the optimal primer pair. Furthermore, we observed that fluorescence signal intensity increased with rising ssDNA concentrations in the CRISPR-Cas12a system, whereas the test line (T line) signal on lateral flow strips (LFS) was stronger at lower ssDNA concentrations. This divergence arises from the distinct signal generation mechanisms of the two detection formats. In fluorescence detection, the ssDNA acts as a substrate for Cas12a trans-cleavage; higher ssDNA concentrations lead to increased fluorescence emission upon cleavage. In LFS detection, however, excessively high ssDNA concentrations result in an abundance of intact ssDNAs competing with the cleaved products for limited binding sites on the test line, thereby suppressing color development. Consequently, careful ssDNA concentration optimization is essential to balance CRISPR-mediated cleavage efficiency and LFS signal output (29). When comparing the fluorescence detection system and LFS, a notable difference in sensitivity was observed (10^1^ copies/μL vs. 10^4^ copies/μL). Despite this, LFS offers the advantage of field-adaptable operation, making it more suitable for rapid field screening. Notably, the 104 copies/μL detection limit of the LFS format remains practically sufficient for early detection of *B. bovis* infection. This threshold aligns well with the clinically relevant parasitemia levels encountered in naturally infected cattle. Previous studies have demonstrated that nested PCR assays targeting the *SBP2* gene could detect parasitemia as low as 10^−8^ % and PCR-ELISA methods achieved detection of approximately 2.4 × 10^−8^% parasitemia, equivalent to one infected erythrocyte per 2 mL of blood. As demonstrated by our clinical validation where LFS correctly identified all true-positive field samples (Supplementary Figure 5), yielding 100% concordance with conventional PCR. This sensitivity is practically sufficient for field screening applications, even though it is lower than that of the fluorescence readout. The LFS assay with a detection limit of 104 copies/μL is practically valuable for field screening in resource-limited regions. Unlike lab-dependent fluorescence detection, it enables simple, equipment-free on-site testing despite lower analytical sensitivity. Future work will focus on optimizing the LFS ssDNA sequence and concentration to further enhance its performance, providing an even more reliable solution for point-of-care diagnosis of bovine babesiosis. The observed 1,000-fold sensitivity gap between the fluorescence readout (10^1^ copies/μL) and the LFS format (10^4^ copies/μL) warrants further mechanistic consideration. This disparity is not unique to our system and has been consistently documented across multiple CRISPR-based diagnostic platforms. For example, the RPA-CRISPR/Cas12a assay for *Brucella melitensis* achieved a fluorescence detection limit of 1 copy/μL, while the LFS counterpart reached only 10 copies/μL. Similarly, an ERA-CRISPR/Cas12a system for *Mycobacterium tuberculosis* reported sensitivities of 9 copies/μL for fluorescence and 90 copies/μL for LFS, respectively. Several interconnected factors account for this inherent sensitivity discrepancy. First, fluorescence detection operates as a real-time kinetic measurement, wherein the signal accumulates progressively as Cas12a continuously cleaves the fluorophore-quencher reporters throughout the entire reaction period. This temporal integration allows for the detection of even low-level cleavage events. In contrast, LFS provides an end-point colorimetric readout that depends on the accumulation of a sufficient quantity of cleaved reporter to generate a visually discernible test line, inherently requiring a higher threshold of activated Cas12a molecules. Second, the visual interpretation of LFS results by the naked eye is intrinsically less sensitive than instrument-based fluorescence quantification, as human vision cannot reliably distinguish subtle color differences below a certain intensity threshold, particularly in low-target scenarios. Third, the performance of LFS is critically influenced by the stoichiometric balance between the cleaved and intact reporters competing for limited capture sites on the test line; an excess of intact ssDNA molecules can competitively inhibit the binding of gold-labeled cleaved products, thereby attenuating signal development. This competitive mechanism, as previously noted by Lin et al. (30), explains why lower ssDNA concentrations paradoxically yield stronger test line signals in LFS, whereas higher concentrations favor fluorescence output. Consequently, the optimal ssDNA concentration for LFS represents a compromise between providing sufficient cleavage substrate and avoiding competitive suppression, which inevitably constrains the ultimate sensitivity of this format. Given these inherent limitations, the 10^4^ copies/μL detection limit of our LFS method remains pragmatically acceptable for field-deployable screening applications, where the trade-off between analytical sensitivity and instrument-free operability is often practically justified. Future efforts directed at optimizing reporter design (e.g., employing multi-biotin labeling or adjusting the length and sequence of the ssDNA) and lateral flow membrane composition may help further bridge this sensitivity gap.

It should be acknowledged that the number of clinical samples evaluated in this study (*n* = 16) is relatively modest. This limitation primarily reflects the inherent difficulty of collecting field-confirmed positive bovine blood samples in the study region, where natural infection rates vary considerably across herds and seasons. Furthermore, carrier animals typically exhibit low parasitemia, making them clinically inconspicuous and difficult to identify without prior molecular screening. Despite this limitation, the complete concordance with conventional PCR among the tested samples provides a solid foundation for further multicenter and longitudinal validation studies. Ongoing efforts are underway to expand sample collection across diverse geographic origins and seasonal time points to more comprehensively validate the assay's clinical utility. In the present study, the assay was compared only with conventional PCR. While conventional PCR is widely used for routine molecular diagnosis of bovine babesiosis in endemic regions, we acknowledge that quantitative PCR (qPCR) and nested PCR assays offer higher analytical sensitivity and are increasingly recognized as reference methods for *Babesia* detection. Future studies will incorporate qPCR as a comparator to more rigorously evaluate the diagnostic performance of our ERA-CRISPR/Cas12a assay, particularly for detecting low-grade infections in carrier animals.

To further align this detection method with practical applications, subsequent research may consider optimizing several key steps. For instance, the use of commercial rapid DNA extraction kits [e.g., magnetic bead-based methods; (31)] or the addition of proteinase K to whole blood samples (32) could replace time-consuming and toxic traditional phenol-chloroform extraction, enabling rapid DNA purification from anticoagulated blood or tick samples within minutes. Although isothermal amplification techniques such as ERA offer high sensitivity, they are prone to false-positive results, primarily due to aerosol contamination and non-specific amplification (e.g., primer-dimer formation). To address this issue, we implemented a multi-pronged strategy: (1) incorporation of the Uracil-DNA Glycosylase (UDG) system: By adding dUTP and UDG enzyme to the reaction system, products contaminated from previous amplification experiments can be effectively degraded, thereby preventing false positives (33); (2). closed-tube detection: The CRISPR-Cas12a reaction solution and ERA amplification products were mixed and reacted within the same sealed tube cap, or an integrated lateral flow dipstick device was used to avoid aerosol contamination resulting from tube-opening operations; (3). design of highly specific crRNA: Carefully designed crRNA ensures that Cas12a recognizes only the true target sequence rather than non-specific amplification products, providing a second layer of specificity assurance through CRISPR technology (29). Through these combined strategies, the reliability of the detection results is substantially enhanced, thereby increasing the practical utility of the method in real-world diagnostic settings (34–36).

The assay demonstrates comparable diagnostic performance to conventional PCR in the tested sample set, while offering advantages in terms of simplified instrumentation, shorter turnaround time (The nucleic acid test only takes 40 min, vs. 2–3 h for PCR), and equipment-minimal visualization via LFS. These features make it particularly suitable for field-deployable screening in resource-limited settings. The target *SBP2* gene selected in this study encodes spherical body protein 2, a molecular marker uniquely possessed by *B. bovis*. Genomic data and validated molecular assays demonstrate that *Babesia bigemina* lacks homologous *SBP2* sequences, and established SBP2-based PCR methods exhibit absolute specificity for *B. bovis* without cross-recognition of *B. bigemina* (20). This study performed multiple sequence alignment on *SBP2* sequences of isolates from the Philippines, South Africa, Tanzania, and Thailand, and showed an overall consistency of 95.93%. This intrinsic species specificity of the *SBP2* locus endows our ERA-CRISPR/Cas12a system with reliable ability to specifically identify *B. bovis*.

## Data Availability

The original contributions presented in the study are included in the article/Supplementary material, further inquiries can be directed to the corresponding authors.

## Supplementary material

Supplementary Table S1 The primer sequence used in this experiment.

Supplementary Table S2 ERA system.

Supplementary Figure S1 ERA-PAGE method for screening primers.

Supplementary Figure S2 ERA-PAGE method for screening amplification time.

Supplementary Figure S3 ERA-PAGE method for screening amplification temperature.

Supplementary Figure S4 PCR method for detecting DNA sample results.

Supplementary Figure S5 Detection of *Babesia bovis* in 16 clinical samples using the ERA-CRISPR/Cas12a assay.
