# Supplementary material for: Development of an ERA-CRISPR/Cas12a-based visual diagnostic assay for bovine babesiosis
Source: Front Vet Sci. 2026 Aug 21;13:1917943. doi: 10.3389/fvets.2026.1917943 (PMC13541529; doi:10.3389/fvets.2026.1917943)
Supplement: Supplementary file 1 [file Table_1.docx]

Table 1. The primer probe sequence used in this experiment

| Sequence name | Sequence（5'-3'） | Amplified fragment length（bp） |
| --- | --- | --- |
| SBP2-F1/R1 | F: GATTGATGTATCCAGGTTTGATAATAGTTC  R: ACAAGCACGCCACTAGATTCATAAATAA | 180 |
| SBP2-F2/R2 | F: GGCACAGCACATACTGGACCCTTCATACC  R: ACAAGCACGCCACTAGATTCATAAATAAC | 261 |
| SBP2-F3/R3 | F: TACTGGACCCTTCATACCGCAATGTTCGTC  R: GTTACAAGCACGCCACTAGATTCATAAATAA | 253 |
| PCR*-B.bovis* primer | F: TCAACAACGTACTCTATATGGCTACC  R: CTACCGACCAGAACCTTCTTCACCAT | 298  (Figueroa et al. 1993) |
| crRNA1 | UAAUUUCUACUAAGUGUAGAUCGUACCCCGAACAAGUCGUCAGA | / |
| crRNA2 | UAAUUUCUACUAAGUGUAGAUAGUUGGUAACUCUGACGACUUGU | / |
| ssDNA | 6-FAM-TTATT-BHQ1 | / |
| ssDNA | 6-FAM-TTATT-Biotin | / |

Table 2. ERA system

| Reagent | Liquid addition amount (μL) |
| --- | --- |
| Primer-F | 2.5 |
| Primer-R | 2.5 |
| ddH_2_O | 21 |
| Dissolving agent | 20 |
| Activator | 2 |
| Sample | 2 |
| Total volume | 50 |

In order to save the cost of detection reagents, we prepared one tube of ERA freeze-drying enzyme reagent and divided it into two tubes at a 1:1 ratio, with one tube being 25 μL.

Figure. 1. ERA-PAGE method for screening primers.

Figure. 2. ERA-PAGE method for screening amplification time.

Figure. 3. ERA-PAGE method for screening amplification temperature.

Figure. 4. PCR method for detecting DNA sample results.

Figure 5. Detection of *B. bovis* in 16 clinical samples using the ERA-CRISPR/Cas12a assay.

(A, B) Fluorescence intensity results of 16 clinical samples detected by the ERA-CRISPR/Cas12a fluorescence system; (C, D) Detection results of 16 clinical samples using the ERA-CRISPR/Cas12a lateral flow system.
